# Plant Growth and Soil Water Content Changes under Different Inter-Row Soil Management Methods in a Sloping Vineyard

**DOI:** 10.3390/plants12071549

**Published:** 2023-04-04

**Authors:** Ágota Horel, Tibor Zsigmond

**Affiliations:** 1 Institute for Soil Sciences, Centre for Agricultural Research, Eötvös Loránd Research Network, Herman O. St. 15, 1022 Budapest, Hungary; 2 National Laboratory for Water Science and Water Security, Institute for Soil Sciences, Centre for Agricultural Research, Herman O. St. 15, 1022 Budapest, Hungary; 3 Doctoral School of Environmental Sciences, ELTE Eötvös Loránd University, Egyetem Square 1-3, 1053 Budapest, Hungary

**Keywords:** grapevine, NDVI, leaf chlorophyll, LAI, tillage, cover crop, grass

## Abstract

The main objective of this study was to investigate soil–plant–water interactions based on field measurements of plant reflectance and soil water content (SWC) in different inter-row managed sloping vineyards. The following three different soil management applications were studied: tilled (T), cover crops (CC), and permanent grass (NT) inter-rows. We measured SWCs within the row and between rows of vines. Each investigated row utilized 7 to 10 measurement points along the slope. Topsoil SWC and temperature, leaf NDVI and chlorophyll concentrations and leaf area index (LAI) were measured every two weeks over the vegetation period (May to November) using handheld instruments. We found that management method and slope position can significantly affect the soil’s physical and chemical properties, such as clay or soil organic carbon contents. Cover crops in the inter-row significantly reduced average SWC. The in-row average topsoil SWCs and temperatures were lower in all study sites compared to the values measured in between rows. Significantly higher SWCs were observed for the upper points compared to the lower ones for CC and T treatments (58.0 and 60.9%, respectively), while the opposite was noted for NT. Grassed inter-row grapevines had significantly lower leaf chlorophyll content than the other inter-row managed sites (*p* < 0.001). The highest average leaf chlorophyll contents were observed in the T vineyard (16.89 CCI). Based on slope positions, the most distinguishable difference was observed for the CC: 27.7% higher chlorophyll values were observed at the top of the slope compared to the grapevine leaves at the bottom of the slope (*p* < 0.01). The leaf NDVI values were not as profoundly influenced by slope position in the vineyard as the chlorophyll values were. For overall LAI values, the T treatment had significantly lower values compared to NT and CC (*p* < 0.001). Moderate correlations were observed between NDVI and LAI and soil nitrogen and carbon content. In general, we found that both inter-row management and slope position can significantly influence soil parameters and affect plant growth, and consequently can accelerate plant stress under sub-optimal environmental conditions such as prolonged drought.

## 1. Introduction

Soil–plant–water monitoring allows information to be rapidly obtained on plant stress caused by water or nutrient deficiencies in soils. Viticulture is an important agricultural sector in many countries, and winemaking and wine itself have major impacts on economic, environmental, and social sectors. With changes in climatic conditions associated with rising air temperatures and decreasing amounts of precipitation forecasted for many regions [1], it is becoming increasingly important to study changes in soil water content and their relationship with the soil-vegetation systems.

Changes in the soil–plant–water system can be strongly influenced by a number of natural and anthropogenic factors. Such factors include the natural environment in which the vegetation is located and the soil management practices used to enhance crop or fruit yield. Soil moisture can be affected by vegetation or land use types [2,3], topography or slope direction [4,5], climate [6], latitudes [7], soil physical and chemical properties [8], or soil management methods [9], etc., each with additional sub-categories that further influence the hydrological processes in the area. In the present paper, we mainly focus on two major factors of soil–plant–water systems, namely inter-row management and slope positions. To obtain a more complete picture of the influencing factors of the system, we used soil physics, soil chemistry, and plant parameters measured during the study. In the light of the accelerated changes in soil hydraulic processes associated with climate change [10], it is vital to know how a particular ecosystem is affected by these changes. This is especially true for the less studied Pannonian regions, where a further decrease in rainfall amounts is expected in the future during the growing season [11].

Inter-row soil management in vineyards has multiple purposes, such as reducing soil erosion, reducing the need for the addition of inorganic fertilizers, and providing safe passage for utility vehicles. Inter-row soil management practices can greatly influence soil water balance elements such as evaporation or infiltration rates, hence, in arid and semi-arid regions optimizing water use is crucial for sustainable viticulture. Inter-row soil cultivation in vineyards often consists of tillage, the planting of cover crops, or permanent vegetation such as grassed inter-rows. Soil tillage, which is normally shallow tillage with a depth of 10–20 cm, can help rainwater to infiltrate deeper into the soil [12], resulting in more soil water being available for the plants in the root zone. However, surface characteristics, such as soil cracks, holes, and surface sealing, can also play an important role in the extent of water infiltration. Tillage results in a bare soil surface, which allows greater soil erosion after greater rainfall, especially in sloping environments. On the other hand, grass cover or cover crops can reduce soil erosion [13]. Cover crops are often planted between grapevine rows, providing many benefits to the soil and consequently to the crop, such as enabling nitrogen fixation (e.g., alfalfa), adding green manure to the soil, or reducing soil erosion. Cover crops in vineyards can also restore organic matter and can increase biodiversity and fertility in degraded soils [14]. During prolonged drought, however, grapevine roots and the cover crop roots can compete for the plant available soil water, and this can result in water-related plant stress. Another benefit of grass cover in the inter-rows is that it can provide a safer passage for machinery carrying out plant management operations such as trimming or harvesting [15]. However, frequent machine traffic can have a major impact on the physical properties of the soil [15,16], and consequently its water regime. Inter-row cultivation methods have numerous purposes, that range from making grapevine farming and management easier to increasing fruit production and quality. However, the subsequent effects on soil temperature and moisture in the grapevine root zone and the inter-row soils are poorly understood. This is particularly relevant because of the expected climate change-related accelerated soil deterioration and the subsequent plant responses, or changes in vineyard ecosystems.

Vineyards are often planted in sloping environments, ranging from moderate to very steep hillsides. Soil cultivation under these conditions can be challenging, especially in terms of soil erosion, and therefore reducing soil loss is crucial. An area’s topography can greatly affect the spatial heterogeneity of soil chemical parameters such as organic matter content [17,18], and physical parameters such as soil particles and aggregates [19,20]. Therefore, spatially heterogeneous chemical and physical properties of soil can significantly influence SWC and plant growth. These differences in soil parameters within a vineyard site can affect the grape quality and quantity [21], therefore, spatially distributed studies can provide crucial information on the current state of soils. Soil redistribution processes in sloping vineyards can cause soil deterioration, as soil aggregates can be transported to the lower portion of the slope. In addition the available soil depth for root growth changes, especially when a relatively shallow soil layer is located above the bedrock [2]. Slope positions not only differ in soil chemistry and physics, due to erosion processes, but their radiation exposure can also be different as well, causing further changes in grape quality [22].

Vegetation indices can provide vital information on the current state of a plant, and can be used to study how plants respond to stressful environmental conditions [23]. Monitoring aboveground vegetation properties such as leaf chlorophyll content, the normalized vegetation index (NDVI), or leaf area index (LAI) can provide information on plant health, canopy structure, and photosynthetic activities [24,25,26]. Plant NDVI relates to plant greenness and density and has been widely used as an indicator of photosynthetic activity, light absorption, and canopy structure [24]. NDVI values have been used in a number of studies to estimate or correlate plant traits such as net primary production [27,28], absorbed photosynthetically active radiation [26,28,29], LAI [26,30,31], plant biomass [32,33], and evapotranspiration [34,35]. However, these studies have reported varying findings relating to how well the values of specific plant species correlate with certain soil types and environmental parameters.

As climate change and its effect on viticulture and vine production are imminent, new solutions are necessary to lessen any adverse effects on the grapes. Therefore, we hypothesized that vegetative inter-row management could significantly affect soil hydrology, and consequently plant growth and plants’ response to water stress. The aim of the study was to determine (1) how different inter-row soil management methods affect soil water content in and between grapevine rows, and (2) how vegetation indices change in relation to inter-row management and slope position.

## 2. Materials and Methods

### 2.1. Site Description

The study area is located in a small, agriculturally dominated catchment (21 km^2^) of the Csorsza stream feeding Lake Balaton in Hungary. This region has a continental climate with Mediterranean and Oceanic influences. The summer season is hot and dry, while the winter season is cold [36]. The study site is a moderately rain deficient area. The mean annual precipitation is around 578 mm, and the average wind speed is 2.8 m s^−1^ (2016–2022). The annual precipitation sum was 422.8 mm in 2022, which is 36.6% lower than the average rainfall during the last 7 years. The average air temperature is around 10.76 °C (2016–2022). During the vegetation period, the mean air temperature is around 15.5 at the research site [36], and at the study site it was 18.1 °C between May 2022 and November 2022.

The research site has a long history of viticulture, mainly cultivated on medium to steep slopes, and soil erosion is therefore a major problem. Due to its high susceptibility to erosion, the research site and its surroundings have collected data on soil moisture, soil temperature, and general meteorological data (e.g., precipitation, air temperature, wind speed and direction, humidity, air pressure, solar radiation) since 2015. Preliminary meteorological data show decreasing trends in annual precipitation amounts from the long-term average [2].

The present experiment was carried out in 2022, during the vegetation period (end of May to early November) of the grapevines. The cultivated grape is a white vine variety (*Vitis vinifera*). Annual light topping is carried out at the study site and weed control is performed when necessary. Harvest is normally carried out from late September to late October. At the study site, all soils and plants are managed in the same way, including applying the same amount and type of fertilizer (organic manure) and trimming plants at the same time. Therefore, at this study site, the effects of inter-row management and slope position can be investigated accurately.

The soil of the study site is generally considered as loess, which is fairly homogeneous after the top few centimeters of topsoil. The soil in the area is generally Cambisol or Luvisol, developed from loess [37].

**Figure 1:**
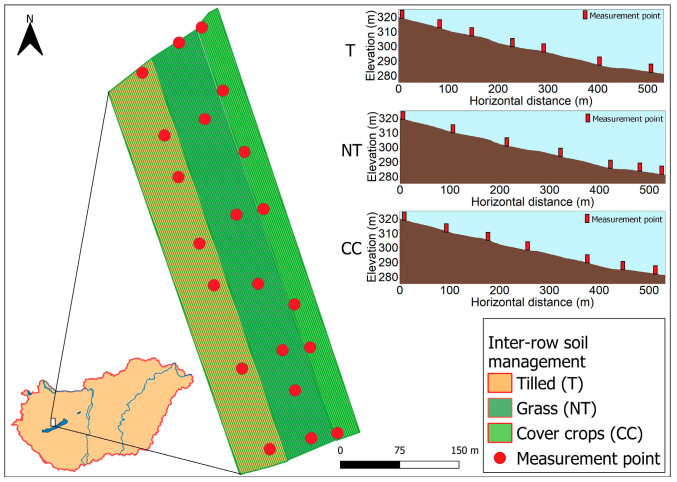
Area of the study site showing locations of measurement points and slope inclines of the different inter-row managed parcels. CC refers to cover crops inter-row, NT refers to no-tillage performed with permanent grass-covered inter-row, and T refers to the shallow tillage inter-row cultivation method.

Three grapevine rows with the following three inter-row cultivation methods were used: (i) tilled inter-row with no vegetation present (T); (ii) inter-row with cover crops (CC) sown (i.e., red clover and alfalfa mix); and (iii) grassed inter-row (NT). The row lengths were between 534 and 553 m. The total area of the CC site is 1.78 ha with a 7.58% gradient, the NT site is 3.50 ha with a 7.29% gradient, and the T site is 3.39 ha with a 7.27% gradient. The distance of the NT row from the T row is approximately 55 m, and to the CC row is approximately 32 m (Figure 1). All of the studied vineyard slopes are organic and non-irrigated sites.

To investigate the effects of soil redistribution processes on the SWC and plant parameters, each slope was divided into lower and upper portions. Therefore, slope positions are defined as the first and last measuring points’ data along the inclines, representing the upper (U) and lower (L) parts of the slopes.

**Figure 2:**
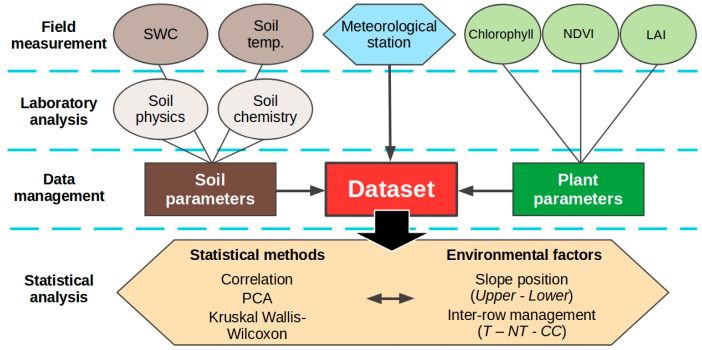
Flow diagram for experimental data collection and analysis.

The general experimental data collection and analyses are summarized in Figure 2, and described in detail below.

### 2.2. Soil Sampling and Analyses

Soil samples for chemical and physical analyses were collected in triplicate from the top 20 cm of the soil layer. Samples were homogenized, sieved (<2 mm), and analyzed for soil texture and total nitrogen, NH_4_^+^-N, NO_3_^−^-N, soil organic carbon (SOC) content, and pH_(H_2_O)_. The amount of total nitrogen was determined using the modified Kjeldahl method (ISO 11261:1995). The amount of SOC was measured based on the Tyurin method by wet digestion. The soil pH was measured using a MultiLine P4 (WTW Multi 350i) pH and electrical conductivity meter in 1:2.5 soil-to-water suspensions. Soil element concentrations are reported as mg kg^−1^ dry weight soil.

### 2.3. Soil Water Content and Temperature

Soil water content was measured at the same points as the plant measurements from all three inter-row managed slopes. Triplicate measurements were taken in a row (under the canopy—IR) and triplicate measurements between rows (inter-row managed bare soil, grassed, or cover cropped areas—BR). SWC was measured using a Hydrosense II (Campbell Scientific) handheld instrument, averaging the top 12 cm of soil water content. Soil temperatures were measured by digital soil thermometers (Grasstec Group) from the top 2 cm of the soil.

### 2.4. Plant Measurements

Plant measurement points were chosen at the beginning of the vegetation period, and each point was approximately four meters in length. All points were marked and we used the same points at each measuring time.

We determined the leaf area index (LAI) of the three inter-row managed grapevines using an AccuPAR LP 80 (Meter Group) instrument, which computes LAI from above and below canopy readings of PAR and leaf angle distribution parameters. LAI measurements were taken only at the top and bottom of each slope, where the grapevines were measured with 40 replicates and averaged.

The chlorophyll content of the grapevine leaves was measured between May and October. We used an Apogee MC-100 instrument, where the values were given in chlorophyll content index (CCI) for general measurements. The instrument used 63.6 mm^2^ measurement area per sample. This instrument calculates the chlorophyll content from the ratio of optical transmission at 931 nm (NIR wavelength) to the optical transmission at 653 nm (red wavelength, Equation (1)).

(1) $$\mathrm{CCI}\;=\;\frac{\%\;\mathrm{Transmittance}\;\mathrm{at}\;931\;\mathrm{nm}}{\%\;\mathrm{Transmittance}\;\mathrm{at}\;653\;\mathrm{nm}}$$

At all study points, we took 15–20 replicate measurements and averaged them. The chlorophyll measurements were taken randomly from grape leaves within the canopy.

Grapevine leaf NDVI was measured using a PlantPen model NDVI 310 (Photon Systems Instruments) device between June and October. This device compares reflected light at two distinct wavelengths of 635 and 760 nm. The NDVI values were calculated based on radiance using the following equation:

(2) $$\mathrm{NDVI}\;=\;\frac{{\mathrm{Nr}/\mathrm{Ni}\;}_{760\;\mathrm{nm}}{\;-\;\mathrm{Nr}/\mathrm{Ni}\;}_{635\;\mathrm{nm}}}{{\mathrm{Nr}/\mathrm{Ni}\;}_{760\;\mathrm{nm}}{\;+\;\mathrm{Nr}/\mathrm{Ni}\;}_{635\;\mathrm{nm}}}$$

Similarly to the leaf chlorophyll measurements, 15 to 20 replicates per point were measured within a four-meter length and averaged. The NDVI measurements were taken randomly from grape leaves within the canopy.

### 2.5. Statistical Analysis

The effects of slope position and inter-row management (tillage, cover crops, or grassed inter-row) on soil water content and plant parameters (leaf chlorophyll content and NDVI) were analyzed using the nonparametric Wilcoxon test and Kruskal–Wallis ANOVA for non-normally distributed datasets. For statistical purposes, we used the same number of measurements for each row, e.g., 7 NDVI and 7 leaf chlorophyll for T, CC, and NT rows. Pearson’s correlation coefficient (*r*) was used to calculate the linear correlation between the soils’ physical and chemical characteristics and plant parameters. Principal Component Analysis (PCA) multivariate analysis was applied to explore the factor pattern of the selected soil and plant parameters. All statistical calculations were performed using the software package R (ggplot2 for the numbers, Hmisc for the correlations, ggpubr for the Wilcoxon test, and the multcomp and multcompView packages for the statistical letters; R Core Team, Version 4.0.2, Vienna, Austria). Statistical significance of the results was determined at *p* < 0.05.

## 3. Results

### 3.1. Comparison of Soil Physical and Chemical Properties between Treatments and Slope Positions

Soil physical and chemical properties for the different slope positions and inter-row management methods are summarized in Table 1. Based on soil texture, erosion-derived differences are presented. The upper part of the study plots had significantly lower sand and higher clay contents than the lower part (*p* < 0.05); however, when averaging the entire data set for each treatment plot, the soil particle size distributions of the three sites were not significantly different.

Soil pH values were relatively similar, with all data showing slightly basic soil pH. Soil chemical characteristics of the different sites showed that upper slope positions had generally higher total N contents, compared to the lower positions, and that NT and CC upper slope positions had significantly higher total N than did T or any of the lower positions (*p* < 0.05; Table 1). Similarly to the nitrogen content, SOC values were significantly higher in NT and CC upper slope positions, while the lowest SOC was observed in the T slope (Table 1).

### 3.2. Soil Water Content and Temperature Changes during Plant Growth

**Figure 3:**
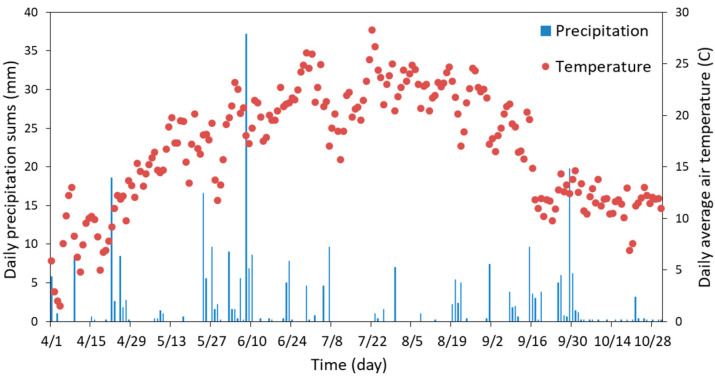
Daily rainfall sums (bars; mm) and daily average air temperature (circles; °C) during the study period.

Below-average rainfall was recorded during the growing season, with a total of 306 mm from April to November (Figure 3), compared to an average of 392 mm during this period over the last seven years [2]. The average air temperature of 16.8 °C was similar to temperatures measured in other years at the study site (16.3 °C) during the vegetation period.

**Figure 4:**
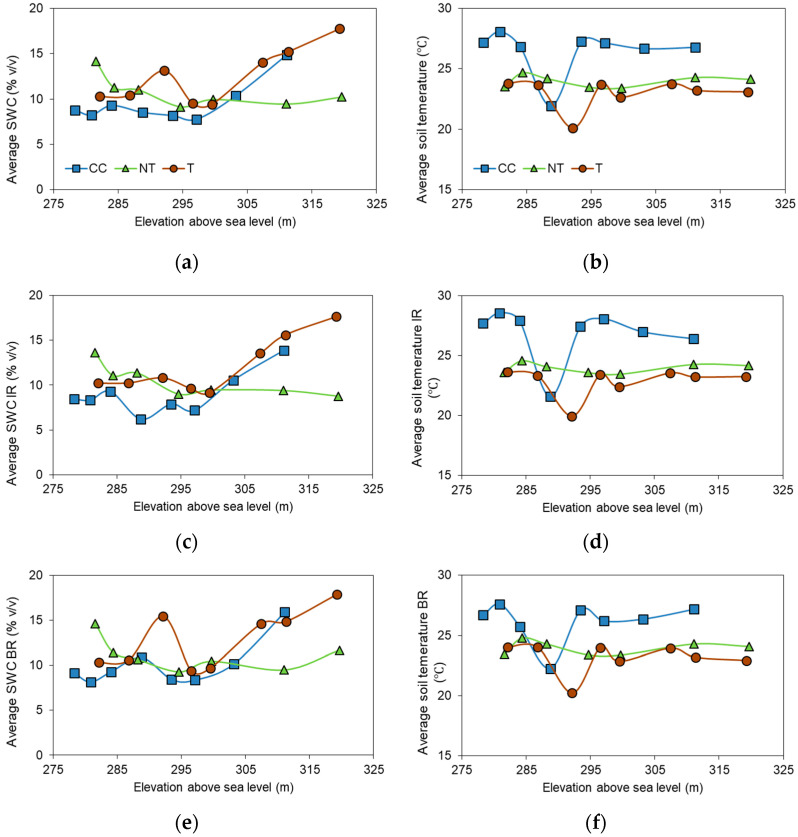
Average soil water content (SWC) and temperature for the different inter-row managed grapevine sites. Average (**a**) SWC, (**b**) soil temperature, (**c**) within row SWC, (**d**) within row soil temperature, (**e**) between row SWC, and (**f**) between row soil temperatures versus elevation above sea level. IR represents values measured within grapevine rows, and BR represents values measured between grapevine rows. n = 39 (IR and BR) and n = 78 total.

Soil water content was measured in the top 12 cm of the soil layer between (BR) and within (IR) grapevine rows. The highest average SWCs were observed for T and the lowest for CC treatments, for both BR and IR measurements (Figure 4a,c,e). The overall SWC was significantly higher for T compared to the other inter-row managed sites (*p* < 0.05), while CC and NT did not show significant differences (*p* > 0.05).

Soil temperatures were the highest in the CC treatment (*p* < 0.001) and the lowest in the T treatment (Figure 4b,d,f). Along the slopes, some outliers were observed. Lower soil temperatures often corresponded to higher SWCs, an effect which is especially distinguishable in the middle part of the transects, between 300 and 400 m from the top of the slopes (Figure 4).

The effect of slope positions on SWC and temperature were also considered in the analyses by comparing data from the top (upper) and bottom (lower) rows. The highest average SWC_IR_ was 17.64% (*v*/*v*) for the T lower point, and the lowest for the CC upper and NT lower points (8.45 and 8.78%, respectively). SWC_BR_ measurements were similar, with the highest for the T lower point (17.64%) and the lowest for the CC upper point (9.12%). Average soil temperatures did not differ significantly between upper and lower slope positions, nor between BR and IR measurements.

### 3.3. Inter-Row Management Effects on Vegetation Indices

**Figure 5:**
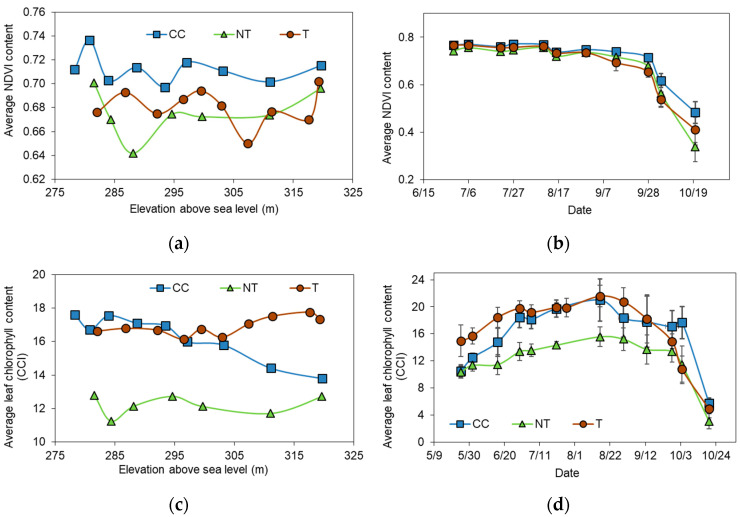
Average leaf (**a**) NDVI over slope position (n = 165) and (**b**) over time (n = 165, ±SD); and (**c**) chlorophyll contents over slope position (n = 195) and (**d**) over time (n = 195, ±SD) for the different inter-row soil managed grapevines.

The plant traits leaf NDVI and chlorophyll concentrations were measured bi-weekly, including the major different plant phenological phases. Therefore, the average values include measurements ranging from bud breaks to after harvest, when leaves were changing colors. Our results showed no clear relationship between elevation and leaf chlorophyll or NDVI values when analyzing all measured data points; however, when only the average values over the vegetation period were considered, some relationships were shown between inter-row managed sites (Figure 5).

The NDVI values were similar among the three different inter-row managed sites (Figure 4a,b). The highest NDVI was observed in the CC and the lowest in the NT treatments (*p* < 0.02). Slope position was not associated with significant differences in leaf NDVI values. The lowest average leaf NDVI was observed for the T upper point (0.676) and the highest for the CC lower point (0.715). Likewise, when studying leaf NDVI changes over time, we observed very similar values between treatments (Figure 5b), and the greatest differences were observed after harvest.

There were distinct differences observed for leaf chlorophyll contents between the different inter-row managed grapevine rows (Figure 5c,d). NT showed significantly lower values, with an average 12.02 CCI, compared to the other inter-row managed grape leaves (16.21 and 16.89 CCI for CC and T, respectively; *p* < 0.001). Leaf chlorophyll values for CC and T were statistically not different (*p* = 0.065). When leaf chlorophyll values within slope positions were analyzed, the most notable difference was observed at the CC site, where the lower point had significantly lower leaf chlorophyll values compared to the upper point (*p* = 0.038). NT and T treatment grapevines did not show significant changes between slope positions (*p* < 0.05). The temporal variation of leaf chlorophyll data is shown in Figure 5d, where NT demonstrates continuously lower values compared to the data collected from the other inter-rows. However, after harvest, the leaf chlorophyll values declined to a similarly low level in all treatments.

**Figure 6:**
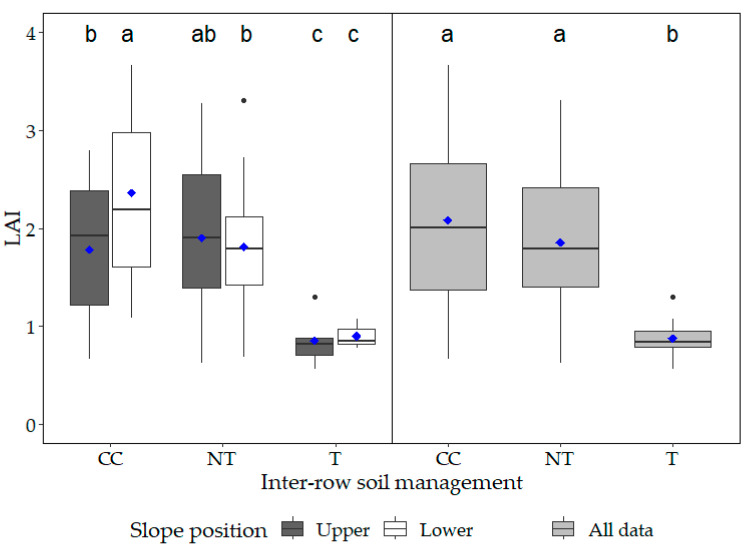
Average leaf area index (LAI) for the different inter-row soil managed grapevines. Each data point represents the median (solid black line), the mean (blue diamond), the upper and lower quartiles, and the minimum and maximum values (whiskers; data plus/minus 1.5 interquartile range). Different letters indicate statistically significant differences between the different slope positions and treatments. (n = 150).

The LAI was measured over the vegetation period for the upper and lower slope positions only. We found that the T site had significantly lower LAI compared to the other sites (*p* < 0.002; Figure 6). The highest LAI was observed for grapes grown in the CC site, and this was significantly higher compared to the other sites (*p* < 0.05). Among slope positions within the same inter-row managed sites, we found that, within the CC treatment, LAI was significantly higher at the lower point compared to the upper (*p* = 0.01; Figure 6).

### 3.4. Relationships between Soil and Plant Properties

**Figure 7:**
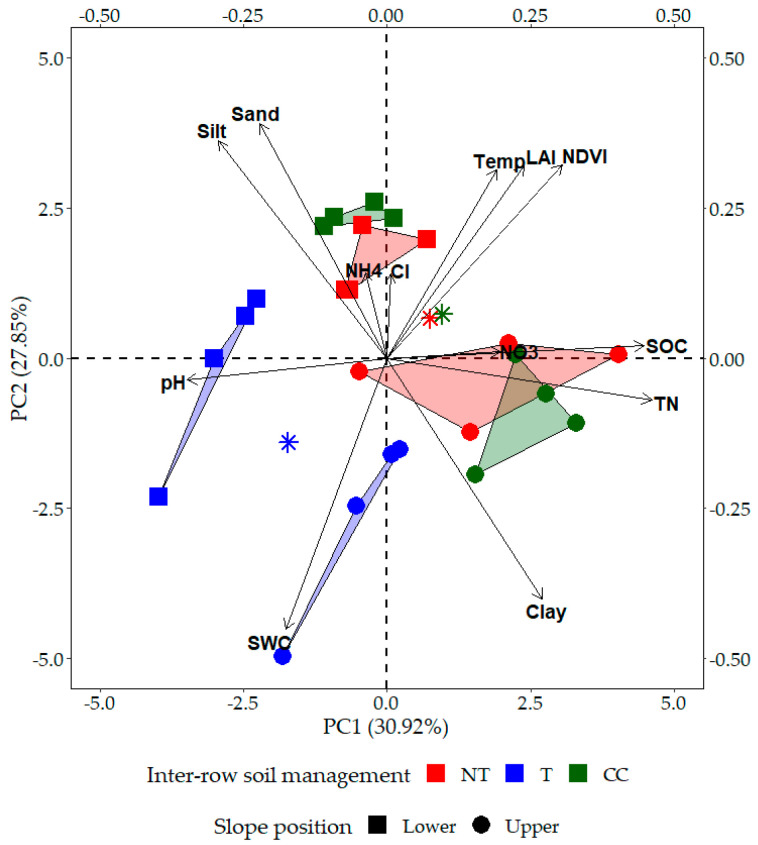
Principal component analysis (PCA) ordination biplot for the three selected slopes with environmental variables (selected soil parameters and plant traits) represented as vectors, and the pairwise representation of the study sites. SWC is the soil water content, Temp is the soil temperature, SOC is the soil organic carbon content, TN is the total nitrogen content, and Cl is the leaf chlorophyll content. Stars represent the study site (inter-row) averages.

We separately investigated the data from three different inter-row managed grapevine plots and the data collected based on slope position. PCA analysis revealed that the first principal component (PC1) accounted for 30.92% of the variation caused by the interaction, while PC2 accounted for 27.85% (Figure 7). The data show clear partitioning of upper and lower slope positions, rather than of inter-row soil management methods. Based on the PCA and Pearson correlation methods of analysis, our results show a negative correlation between leaf NDVI and SWC concentrations (r = −0.60; *p* = 0.003). A significant but weak correlation was observed between NDVI and LAI (r = 0.57; *p* = 0.001) and between leaf NDVI and chlorophyll contents (r = 0.38; *p* = 0.034). Negative correlations were noted between soil temperature and SWC (r = −0.80; *p* < 0.001). Among soil properties, strong correlations were shown between SOC and total N contents (r = 0.96; *p* < 0.001) and between sand and clay contents (r = −0.96; *p* < 0.001). We found positive, significant correlations between total N and clay content (r = 0.61; *p* < 0.001) and negative correlations between total N and pH, sand, or silt (r = −0.53, −0.54, −0.62, respectively; *p* < 0.002).

## 4. Discussion

Published studies have reported that both inter-row management methods and the types of vegetation planted between rows of vineyards have inconsistent effects on soil physical properties such as SWC, on plant traits of the grapevines such as leaf density or canopy structure, and on vegetative growth such as pruning weight or fruit yield [38]. In this study, the effects of inter-row management on soil water content and the plant parameters NDVI, leaf chlorophyll, and LAI were investigated in sloping vineyard rows.

Water stress can cause changes in vine growth and development, such as a reduction in pruning weight [39], photosynthesis, or leaf conductance [40]. In our study, the inter-row soil cultivation methods had significant impacts on both soil properties and plant traits. SWCs of the top 12 cm of the soil layer were the highest for the soil with shallow tillage (T) and the lowest for the cover crop (CC) planted inter-rows. There are several possible explanations for this, one of which is that the water usage was lower by the grapevines in the T treatment compared to the other vine rows. However, this is somewhat unlikely if the vine variety and the age of the vines are fairly similar among treatments. Another reason is that interception and evaporation from the grass leaves and cover crop leaves were high enough to cause the differences in SWC between different inter-rows. Interception is an important factor affecting soil water content, as it can be as high as 70–80% of low intensity rainfall amounts for grass [41]. During the study period, smaller rainfall events were more frequent at the vineyards; therefore, the interception in the CC and NT sites could be substantial and could result in lower water infiltration. Since the soil temperature was significantly higher in the CC rows than the other two study rows, the higher evaporation rate could further decrease the water content of the topsoil. However, it has been also suggested that vegetated soils can retain more infiltrating water than bare soils, suggesting that evaporation from bare soils can be higher than evapotranspiration by vegetated sites [42,43]. Finally, one of the most important reasons for lower SWC in the CC and NT sites compared to bare soil (T) is that both grass and cover crop roots take up water from the soil and these plants are competing with shallow vine roots, and therefore less plant available soil water remains in the upper soil layers. The long root structure of mature grapevines enables them to retrieve water from deeper into the soil matrix [44], while for low fertile vineyard soils or younger vines with less developed root structure, soil moisture deficit due to vegetated inter-row can cause water stress for the plants. Grape root structures are seasonally and spatially non-evenly distributed, and root distribution can change, such as root shedding in dry areas and rapid root growth in moist areas in the soil [45]. The investigated grapevine rows were located on moderately steep slopes (on average 7.27–7.58% inclines with up to 14.5% slope within the rows), where the different levels of vegetation cover could influence the deep percolation or runoff of rainfall. Soil physical and chemical differences among study sites can greatly influence the hydrology of the soils; however, in the present study, the differences in soil texture between sites were less pronounced than the heterogeneity within the same slope. Magdić et al. [46] found similar results, reporting that the upper part of the vineyard slope had higher soil moisture content compared to the lower points. These authors also suggested that higher clay content could be a possible cause. Soil organic carbon content can be directly related to SWC [47] although, in our study, the highest SOC values were observed for the NT and CC upper points. The time of the tillage in vineyards is very important in terms of infiltration and water runoff, as immediately after tillage is performed, an increase in water infiltration can occur [48]; however, over the vegetation period, this effect is expected to diminish. Therefore, the differences in the SWCs in our study were most likely due to the water uptake and interception by the vegetation planted between rows, rather than differences in soil properties.

Plant traits can be used as proxies to estimate plant-related stress due to environmental conditions. When plant growth conditions are below optimal, lower biomass or fruit production can be expected. NDVI is one of the most widely used parameters that can provide valuable information on plant greenness, biomass, and overall leaf health [24,26,49,50]. Sub-optimal soil chemical or physical properties of nutrient or soil water deficiency, or biological stress such as diseases caused by insects or fungi, could result in a decrease in NDVI values. Grape quality is not uniform within a given vineyard, and zones can be determined using NDVI values and fruit load [51]; therefore, the measurement points in the present study that were randomly chosen prior to bud break could enable the inclusion of different plant biomass qualities. In our study, NDVI was significantly higher for CC-managed rows compared to NT, but not for T management, indicating that cover crops can be beneficial for plant development. Similar results were observed by Pornaro et al. [52], where the authors highlighted that the type of vegetation between rows may have affected the NDVI values of grapevines more than the presence of the plant itself. Therefore, the type of cover crop is also an important factor in vineyards, as it can be beneficial or detrimental to the vine’s environment. In our study, NDVI and SWC showed a negative correlation (r = −0.60), which is the opposite of what was expected. Correlations between NDVI and soil moisture can depend on many factors and can result in inconsistent data. Higher SWC can result in higher NDVI values [53], and, based on plant growth, either positive or negative relationships can be produced [54], or the relationship could depend on vegetation cover and soil type [55]. Since only the SWC of the topsoil was taken into account in the present study, incorrect results might have been produced, as the vine can take up water to nourish the plant from deeper soil layers, while the topsoil is drier. While the present study specifically focused on grape leaves, including the inter-row vegetation NDVI and deeper layer SWC in the analysis would allow more accurate inferences.

Pruning or topping of grapevines are often performed to simulate the plants to promote the ripening of fruit branches bearing grapes and therefore, these processes can cause changes in plant development. Leaf chlorophyll content may be sensitive to drought or tillage operations [56], or nitrogen content [25]. In our study, leaf chlorophyll content was significantly higher in the CC and T rows than in the NT row, which is consistent with a study by Griesser et al. [57], where the authors found that soil tillage can increase leaf chlorophyll content. Córdoba et al. [58] also found significantly higher chlorophyll with soil tillage than many vegetated inter-row managed grapevines, and the authors further highlighted the importance of the yearly effects. Since chlorophyll values in grapevine leaves have a high spatio-temporal variability [59], our frequently collected data on whole vine rows have allowed us to evaluate whether changes in plant traits are sudden or uniform over time and whether there are some hotspots with outliers. Our data did show a few spots among the slopes in which data on plant parameters changed suddenly; however, for leaf chlorophyll data, this was not as pronounced as for NDVI. Therefore, the water content and temperature of the topsoil, which were notable for hotspot locations, did not affect leaf chlorophyll. Drought conditions can significantly reduce plant chlorophyll content [60]; however, as mentioned earlier, the longer root structures of the vines could take up water from the deeper soil layers. Chlorophyll values showed a weak but significant correlation with NDVI values; however, temporal variations in correlation coefficients were also noted [61]. During the flowering and fruit maturation period, the relationships between NDVI and chlorophyll are expected to be stronger. Monitoring leaf chlorophyll contents in vineyards can allow the evaluation of their nitrogen status, as these can be directly related [62]. This can be a great tool for farmers to manage fertilizer application, as increased nitrogen levels due to improved nutrient availability from the soil can increase chlorophyll concentrations in the leaves [63]. Our findings also suggests that overall plant growth and health can be monitored by measuring both leaf NDVI and chlorophyll parameters, and that a decline in any of the values can indicate concerns about plant health.

LAI and NDVI also showed a moderate and significant correlation in our study; however, we only measured LAI for the upper and lower point of the grapevine rows. Strong relationships between NDVI and LAI may be expected, as a denser canopy normally indicates healthier and greener leaves, and many studies have found strong connections between these parameters [31,61,64,65]. The weaker relationship demonstrated in our study may be due to the highly variable soil parameters along the slopes (e.g., significantly lower SOC, total nitrogen, and NO_3_^−^-N contents for T), resulting in lower LAI values for the T treatment, while the NDVI values were similar in all grapevine rows. In the present study, LAI was only measured in two positions per slope, therefore, in future studies, more numerous LAI measurements should be taken, to enable stronger conclusions to be drawn.

## 5. Conclusions

The present study investigated the effects of different inter-row management strategies on soil water content, temperature, and the plant parameters of leaf chlorophyll, NDVI, and LAI. We further investigated how slope position affects these soil and plant parameters. We found that, in contrast to the many advantages of planting vine rows with perennial or permanent plants, during prolonged drought conditions, the grass and cover crops might compete for soil water, which can cause water deficiency and stress in vines. We found that SWC differences among treatments were caused by water uptake and interception by the inter-row vegetation, rather than differences in soil physical or chemical properties. For plant traits, we found that slope position can be a more important factor than the type of inter-row soil management for certain plant traits (e.g., LAI), consistent with significant changes in soil chemistry and physics in our study sites due to soil redistribution processes. In future studies, more emphasis should be given to measurements of inter-row vegetation properties during the main plant phenological phases such as bloom, fruit set, veraison, and fruit maturation. As the effects of climate change on soil hydrological processes may further affect plant–soil–water relations, longer-term monitoring is needed in highly affected areas. Currently, there are some research gaps on the extent of the warming environment and the sensitivity of the soil–plant–water system and its components, and their ability to adapt to rapidly changing environmental conditions, therefore further studies should be conducted.

## Figures and Tables

**Table 1:** Physical and chemical characteristics of the soils collected at different slope positions (upper and lower). Sand 2–0.05 mm, silt 0.05–0.002 mm, clay < 0.002 mm particle sizes. SOC represents soil organic carbon values. Different letters indicate statistically significant differences at *p* < 0.05. n = 6; ±SD.

	Vineyard T		Vineyard NT		Vineyard CC	
	Lower	Upper	Lower	Upper	Lower	Upper
Sand (%)	25.4 ± 1.2 c	15.7 ± 0.5 e	32.2 ± 1.0 a	19.0 ± 1.0 d	27.3 ± 1.1 b	14.4 ± 2.3 e
Silt (%)	60.7 ± 1.0 a	47.6 ± 0.6 d	56.5 ± 0.9 b	53.1 ± 1.6 c	60.7 ± 0.9 a	48.6 ± 1.0 d
Clay (%)	13.9 ± 0.2 c	36.7 ± 0.7 a	11.3 ± 0.7 d	28.0 ± 1.3 b	12.1 ± 0.9 d	37.0 ± 3.1 a
Total N %	0.11 ± 0.0 c	0.15 ± 0.0 b	0.15 ± 0.0 b	0.21 ± 0.0 a	0.14 ± 0.0 b	0.23 ± 0.0 a
NH_4_-N mg/kg	5.2 ± 1.3 a	5.2 ± 1.2 a	7.7 ± 4.4 a	6.2 ± 2.2 a	5.8 ± 2.9 a	5.8 ± 3.0 a
NO_3_-N mg/kg	6.4 ± 2.6 cd	4.9 ± 0.6 d	13.3 ± 10.4 bc	19.5 ± 25.0 abc	24.0 ± 11.7 ab	27.9 ± 10.7 a
SOC %	0.7 ± 0.0 d	0.9 ± 0.0 c	1.3 ± 0.2 b	1.8 ± 0.6 a	1.1 ± 0.2 bc	1.8 ± 0.2 a
pH_(H2O)_	8.1 ± 0.1 a	7.9 ± 0.0 bc	7.7 ± 0.2 bc	7.7 ± 0.2 c	7.9 ± 0.2 ab	7.9 ± 0.1 bc

## Data Availability

Not applicable.
